# Transcriptomic and physiological analyses reveal temporal changes contributing to the delayed healing response to arterial injury in diabetic rats

**DOI:** 10.1016/j.jvssci.2023.100111

**Published:** 2023-05-19

**Authors:** Sampath Narayanan, Samuel Röhl, Mariette Lengquist, Malin Kronqvist, Ljubica Matic, Anton Razuvaev

**Affiliations:** Vascular Surgery, Department of Molecular Medicine and Surgery, Karolinska University Hospital and Karolinska Institutet, Stockholm, Sweden

**Keywords:** Diabetic vasculopathy, Rat carotid balloon injury, Restenosis, Vascular remodeling

## Abstract

**Objective:**

Atherosclerosis is a leading cause of mortality in the rapidly growing population with diabetes mellitus. Vascular interventions in patients with diabetes can lead to complications attributed to defective vascular remodeling and impaired healing response in the vessel wall. In this study, we aim to elucidate the molecular differences in the vascular healing response over time using a rat model of arterial injury applied to healthy and diabetic conditions.

**Methods:**

Wistar (healthy) and Goto-Kakizaki (GK, diabetic) rats (n = 40 per strain) were subjected to left common carotid artery (CCA) balloon injury and euthanized at different timepoints: 0 and 20 hours, 5 days, and 2, 4, and 6 weeks. Noninvasive morphological and physiological assessment of the CCA was performed with ultrasound biomicroscopy (Vevo 2100) and corroborated with histology. Total RNA was isolated from the injured CCA at each timepoint, and microarray profiling was performed (n = 3 rats per timepoint; RaGene-1_0-st-v1 platform). Bioinformatic analyses were conducted using R software, DAVID bioinformatic tool, online STRING database, and Cytoscape software.

**Results:**

Significant increase in the neointimal thickness (*P* < .01; two-way analysis of variance) as well as exaggerated negative remodeling was observed after 2 weeks of injury in GK rats compared with heathy rats, which was confirmed by histological analyses. Bioinformatic analyses showed defective expression patterns for smooth muscle cells and immune cell markers, along with reduced expression of key extracellular matrix-related genes and increased expression of pro-thrombotic genes, indicating potential faults on cell regulation level. Transcription factor–protein-protein interaction analysis provided mechanistic evidence with an array of transcription factors dysregulated in diabetic rats.

**Conclusions:**

In this study, we have demonstrated that diabetic rats exhibit impaired arterial remodeling characterized by a delayed healing response. We show that increased contractile smooth muscle cell marker expression coincided with decreased matrix metalloproteinase expression, indicating a potential mechanism for a lack of extracellular matrix reorganization in the impaired vascular healing in GK rats. These results further corroborate the higher prevalence of restenosis in patients with diabetes and provide vital molecular insights into the mechanisms contributing to the impaired arterial healing response in diabetes. Moreover, the presented study provides the research community with the valuable longitudinal gene expression data bank for further exploration of diabetic vasculopathy.

## Clinical Relevance

Vascular interventions causes injury to the arterial wall, which in turn induces a healing response to restore vessel wall homeostasis. However, in patients with diabetes, such interventions lead to exaggerated healing response and defective remodeling. There is a need to understand the molecular mechanisms underlying the defective healing response in diabetes. In this study, ultrasound biomicroscopy, histology, and microarray profiling were used to demonstrate the transcriptional and physiological changes at various timepoints following arterial injury in healthy Wistar and diabetic GK rats. This study also provides a database of longitudinal transcriptional changes for the research community to study vascular healing in diabetes.

**Article Highlights**

• **Type of Research:** Rat model
• **Key Findings:** Vascular healing in response to balloon catheter injury was significantly delayed in diabetic Goto-Kakizaki rats compared with healthy Wistar rats. Microarray profiling of the injured left common carotid artery at each timepoint revealed defects in smooth muscle cell and immune cell signaling.
• **Take Home Message:** Ultrasound biomicroscopy, histology, and microarray profiling provided physiological and molecular insights into the delayed vascular healing in Goto-Kakizaki rats.

The epidemic of diabetes mellitus (DM) is increasing worldwide, and DM is an independent risk factor for morbidity and mortality associated with cardiovascular disease (CVD).^1^^,^^2^ Prevalence of DM increases cardiovascular risk by three- to eight-fold, and more than 30% of patients with acute myocardial infarction have diabetes.^3^ Vascular interventions in patients with diabetes are prone to an increased risk of restenosis. Restenosis is a re-narrowing of the blood vessel caused by an excessive intimal hyperplastic response along with an impaired vascular remodeling. In addition, proinflammatory cytokines such as interleukin-1β, tumor necrosis factor-alpha, and interleukin-6 in patients with diabetes, induce the phenotype switching of smooth muscle cells (SMCs) from contractile to synthetic phenotype.^4^, ^5^, ^6^, ^7^ Despite the use of drug eluting stents, patients with diabetes still have an increased risk of in-stent restenosis and late stent thrombosis.^8^ It is important to understand the molecular changes occurring during intimal hyperplasia in diabetes in order to design new strategies to prevent restenosis in patients with diabetes.

The major effects of uncontrolled hyperglycemia and insulin resistance in diabetes are manifested as microvascular and macrovascular complications. In large arteries, veins, and in the heart, increased release of free fatty acids from insulin resistant adipocytes results in increased reactive oxygen species (ROS) production and dysfunction of endothelial cells, leading to atherogenesis.^3^ In addition to endothelial dysfunction, prolonged hyperglycemia can induce nonenzymatic glycation of the reactive side chains of amino acid lysine on various proteins resulting in advanced glycation end products (AGE). Studies have shown that a receptor for AGE (RAGE) activates inflammatory pathways in several vascular cells such as SMCs, endothelial cells (ECs), and macrophages, creating an atherogenic microenvironment.^9^

The rat common carotid artery (CCA) balloon injury model is a commonly used experimental model to study intimal hyperplasia and restenosis. The model involves a mechanical stretch injury of the arterial wall and denudation of the endothelial lining of the CCA, which induces a healing response characterized by vascular SMC proliferation, extracellular matrix (ECM) accumulation, and rapid reendothelialization.^10^ Using this model, we have previously shown that vascular injury induces dynamic changes to the transcriptomic landscape in the vessel wall and revealed novel mechanisms contributing to healing in male Sprague Dawley rats.^11^ The Goto-Kakizaki (GK) rats are a non-obese insulin resistant model of type 2 diabetes produced by selective inbreeding for a hyperglycemic phenotype.^12^ Because the GK rats were selected for highest normal blood glucose levels, it is important to point out that they represent an ideal model of type 2 diabetes with impaired glucose-stimulated insulin secretion attributed to a polygenic inheritance.^13^ These rats are characterized by normal circulating insulin levels, but a moderate increase in blood glucose levels resulting from insulin resistance, recapitulating the pathophysiology of the human diabetic condition.^14^ Previously, we had incorporated ultrasound biomicroscopy (US) into the balloon injury model in GK rats to study vessel wall healing in diabetes.^15^

Here, we aimed to combine US imaging, histology, and microarray analysis to systematically study the physiological and molecular changes throughout the healing of the vessel wall in the diabetic environment. We compared the transcriptomic profiles of injured arteries from healthy Wistar rats and diabetic GK rats to identify differentially expressed genes, altered pathways, and transcriptional changes along each timepoint throughout the healing process.

## Materials and methods

### Animals and study design

**Fig 1:**
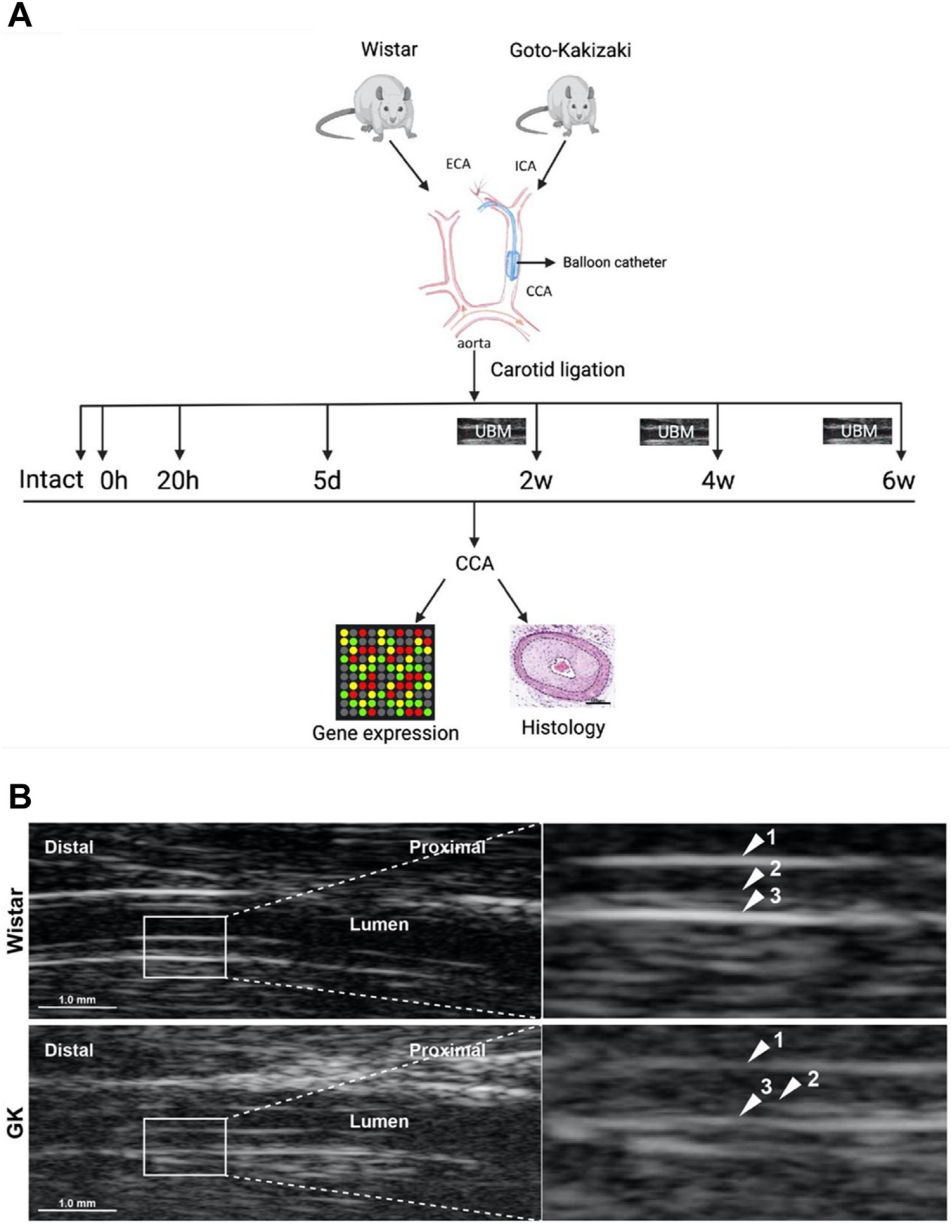
Workflow of the carotid balloon injury model and downstream analysis. **A**, Balloon catheter injury was performed on the left common carotid artery (*CCA*) of Wistar and Goto-Kakizaki (GK) rats and animals were euthanized at different timepoints post-surgery (0 and 20 hours, 5 days, and 2, 4, and 6 weeks) (n = 6-10 for each strain and for each timepoint). Noninvasive ultrasound biomicroscopy (*UBM*) was conducted at 2, 4, and 6 weeks after injury for morphological and physiological evaluation. At all timepoints, microarray profiling and histological analysis was performed. **B**, Representative images from UBM analysis of Wistar and GK rat CCA at 6 weeks. The far-wall portion of the artery has been enlarged for illustration. *Arrows* indicate: (1) lumen-intimal interface; (2) intima-media interface; (3) media-adventitia interface. *ECA*, External carotid artery; *ICA*, internal carotid artery.

Male Wistar were purchased from Charles River (Scanbur Research A/S), and GK rats were bred in-house (generously provided by Prof Claes-Göran Östenson, Karolinska Institutet). Rats were housed in enriched cages at the animal facility and were constantly monitored by professional animal caretakers. The rats (n = 40 for Wistar and n = 40 for GK) were subjected to left common carotid artery balloon injury using 2F Fogarty balloons as previously described^11^ and euthanized at different time points (uninjured, 0 hours, 20 hours, 5 days, 2 weeks, 4 weeks, and 6 weeks) after surgery (6-10 rats per timepoint) (Fig 1, *A*). The timepoints for euthanasia and analysis was selected based on the previous study performed in Sprague-Dawley rats,^11^ where they represent the early, intermediate, and late phases of healing in a normal rat vessel.

Noninvasive morphological assessment was performed using US (Vevo 2100, Fujifilms, Visualsonics Inc; probes: 30-70 MHz, MS700) at 2-, 4- and 6-weeks post-surgery. The US image analysis was performed using an offline imaging software (Vevolab 1.7). US images were used to measure lumen and total vessel diameter as described previously by Röhl et al.^16^

Upon euthanization, both the injured and uninjured (contralateral right CCA) vessels were used for microarray and histological analyses. All animal experiments were approved by the Stockholm Ethical Board (Dnr N181/16; N137/14), and institutional guidelines for proper animal care were followed.

### RNA extraction

RNA was extracted using Qiazol Lysis Reagent (#79306, Qiagen) and purified using the miRNeasy Mini kit (#217004, Qiagen). The RNA concentration was determined using the Nanodrop ND-1000 (Thermo Scientific), and the RNA quality was estimated using a Bioanalyzer capillary electrophoresis system (Agilent Technologies). Total RNA of appropriate quality and integrity (RIN: 8.0-9.8, A260/280: 2.0-2.1, A260/230: 0.3-2.0) was used for microarray profiling with Affymetrix GeneTitan Rat Gene ST v1 arrays.

### Histology

Carotid arteries were fixed in 4% Zinc-formaldehyde for 24 hours and dehydrated in 70% ethanol. Then the tissues were embedded in paraffin blocks. Five-μm sections were obtained using a microtome. The sections were deparaffinized using Histolab Clear and rehydrated in gradually decreasing concentrations of ethanol. After washing, the sections were treated with Bouin's solution (Sigma-Aldrich). The slides were stained with Weigert's iron hemotoxylin (1:1) (Histolab), washed and stained with Biebrich's scarlet-fuschin solution (Sigma-Aldrich). After washing, the sections were stained with phosphomolybdic-phosphotungstic acid solution (1:1+2 volumes of distilled water) (Sigma-Aldrich) and then with Aniline Blue. The sections were washed with 1% acetic acid and dehydrated using increasing concentrations of ethanol. The sections were mounted using Pertex (Histolab) and scanned in an automated SlideScanner system (Scanscope CS).

### Bioinformatic analysis

#### Analysis of the microarray data

Robust multi-array average normalization and batch effect correction of the microarray dataset was performed, and processed gene expression data was returned in log2-scale. Principal component analysis (PCA) was performed using the R software (https://www.r-project.org/). The data was centered and scaled, and PCA was calculated. Differential gene expression analysis was performed using a two-sided multiple *t* tests (assuming equal variances), by comparing Wistar and GK rats for each timepoint. The statistically significant changes in gene expression at each timepoint were represented by a volcano plot. Genes important for vascular healing among the top 20 differentially up- and down-regulated genes were indicated on the volcano plot.

#### Pathway analysis

Pathway analysis was performed using The Database for Annotation, Visualization, and Integrated Discovery (DAVID) online bioinformatic tool (https://david.ncifcrf.gov/). Briefly, a list of up-/down-regulated genes from each timepoint was used to generate gene ontology (GO)-biological processes with an EASE score of 0.0001 and gene count for each pathway of five. The resulting list of pathways were filtered with dispensability less than 0.3.

#### Transcription factor-protein-protein interaction analysis

Transcription factor protein-protein interaction (TF-PPI) analysis was performed using the web-based Enrichr (https://maayanlab.cloud/Enrichr/) and STRING (https://string-db.org/) databases. Significantly up- and down-regulated genes from the pathway analysis were fed into Enrichr to obtain TF-PPIs for each timepoint. The top 10 significantly up- and down-regulated transcription factors from Enrichr were used to generate PPI network using the STRING database. These networks were imported to Cytoscape software (version 3.9.0), where the styles of the nodes were modified according to *P*-value and direction of fold change.

## Results

### Diabetic rats exhibit impaired vascular healing response

**Fig 2:**
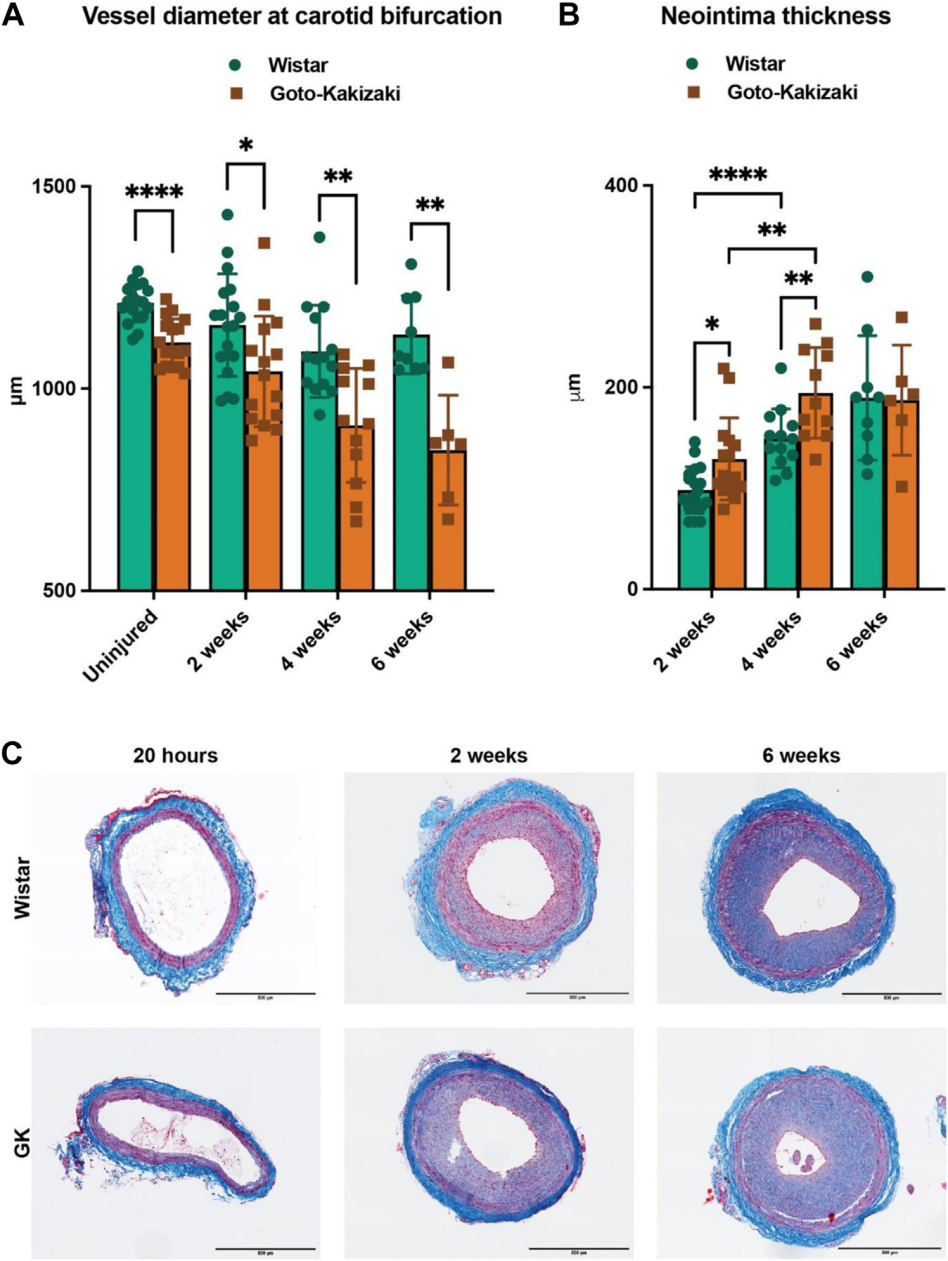
Diabetic rats exhibit impaired vascular healing response following carotid balloon injury. The healing of the artery wall was measured using an ultra high-frequency ultrasound system. Vessel diameter **(A)** was smaller in uninjured Goto-Kakizaki (*GK*) rats and was significantly decreased at 2, 4, and 6 weeks, and neointima thickness **(B)** was significantly increased at 2 and 4 weeks in GK rats compared with Wistar rats. Neointima thickness was also significantly increased at 4 weeks compared with 2 weeks within Wistar and GK rat vessels. Statistical analysis between Wistar and GK at each timepoint was performed using non-parametric Mann-Whitney test; ∗*P* < .05; ∗∗*P* < .01; ∗∗∗*P* < .001; ∗∗∗∗*P* < .0001. **(C)** Representative images for Masson-trichrome staining illustrate an increase in the intimal hyperplasia in GK rats compared with Wistar rats during the late phase of vascular healing (2-6 weeks). Scale bar, 500 μm.

**Supplementary Fig 1:**
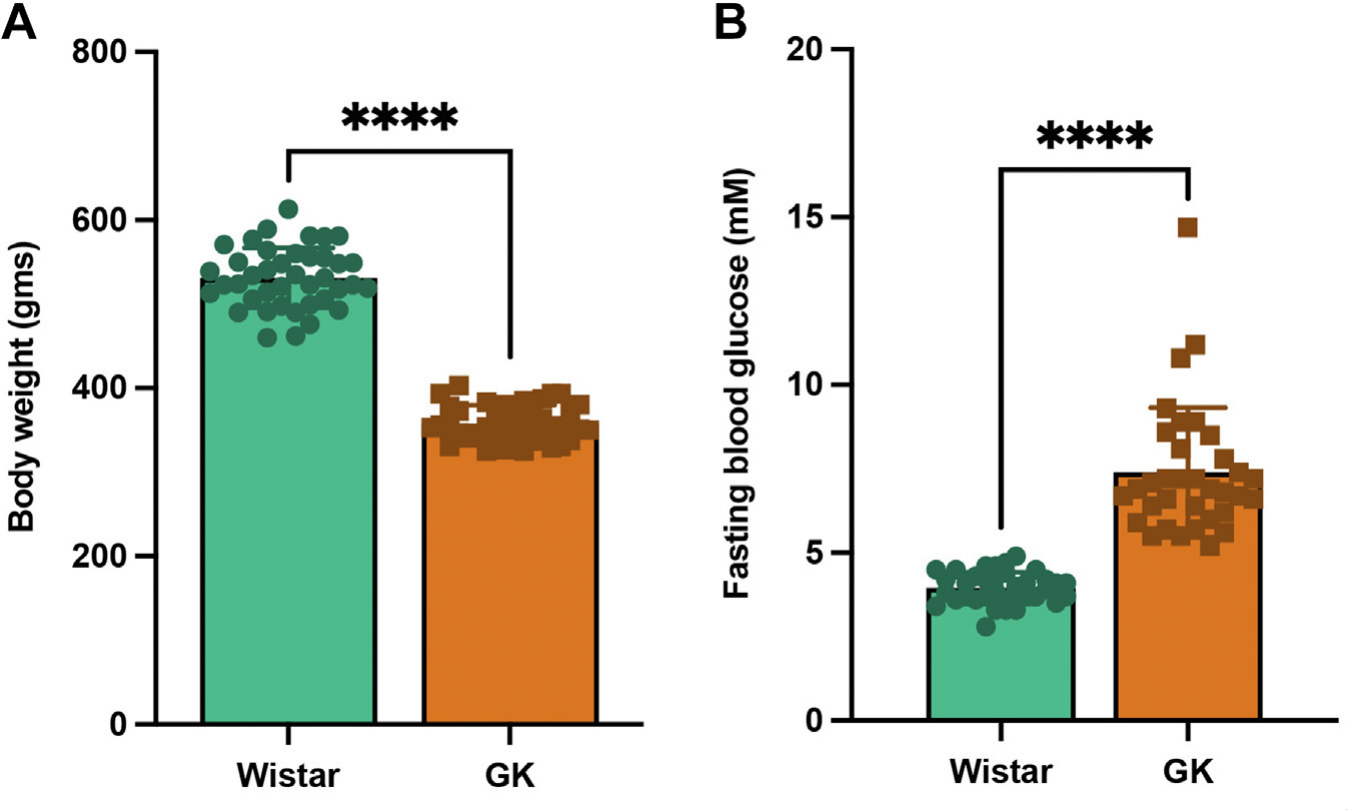
Measurements of body weight **(A)** and fasting blood glucose **(B)** of Wistar and GK rats before the surgery.

Morphological and physiological changes in response to carotid balloon injury in GK and Wistar rats were assessed by US (Fig 1, *B*). We observed that seven arteries from Wistar and 10 arteries from GK rats thrombosed in total at the end of the experiment. The vessel diameter of the intact arteries was significantly smaller in GK rats, as expected, because they correlate with the body weight^17^ (Supplementary Fig 1, *A*). However, the loss of the total vessel diameter was significantly more pronounced in diabetic animals compared with Wistar rats, indicating negative remodeling in these animals at 2, 4 and 6 weeks (Fig 2, *A*). The intima-media thickness was significantly increased in GK rats compared with Wistar rats at 4 weeks after injury (Fig 2, *B*). In parallel with stabilization of the vessel diameter, we could see continuous regrowth of the endothelial layer in Wistar rats.

**Supplementary Fig 2:**
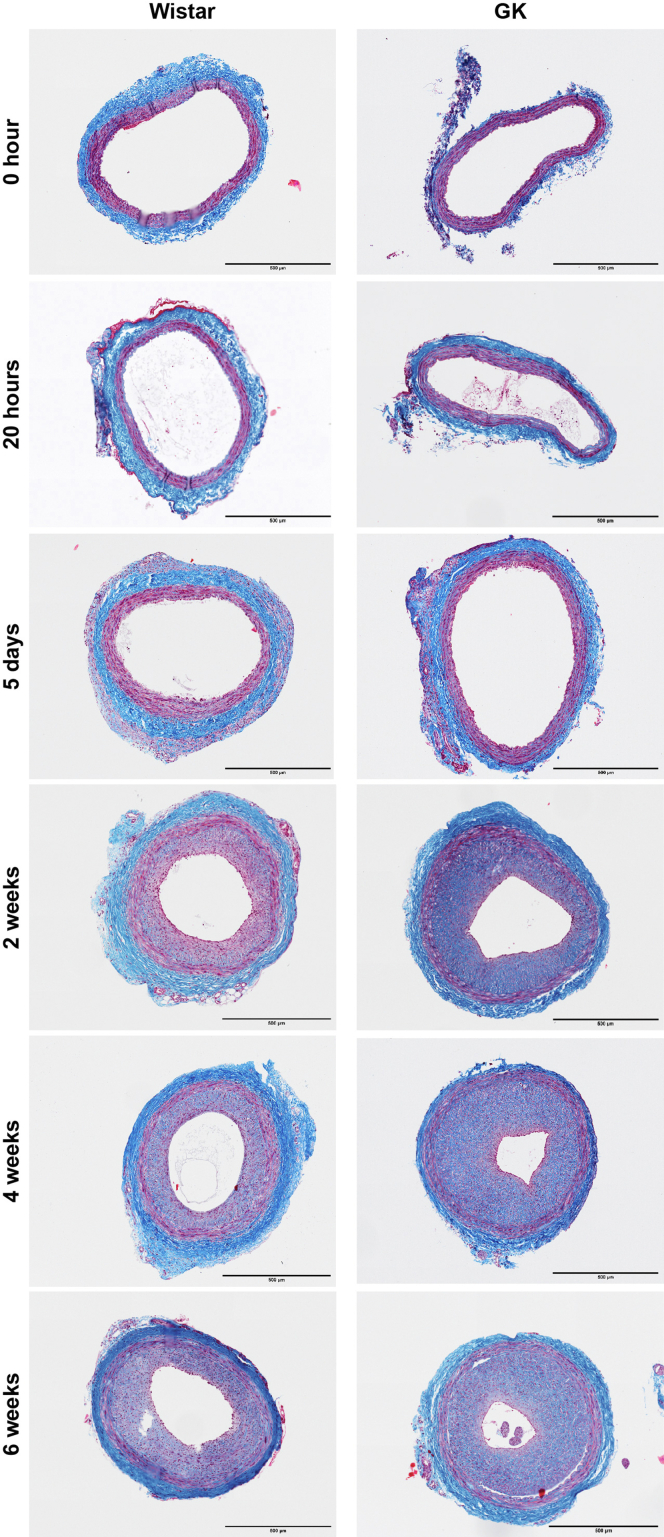
Representative images for Masson-trichrome staining of Wistar and GK rats from uninjured and injured carotid vessels at 0 and 20 hours, 5 days, and 2, 4, and 6 weeks after injury. Scale bar, 500 mm.

Histological analysis, using Masson-trichrome staining, confirmed that the neointimal thickness was greater in GK rats at 2, 4, and 6 weeks (Fig 2, *D* and Supplementary Fig 2). Taken together, these results indicate that vascular wall healing is impaired in GK rats.

### Transcriptomic changes in response to vascular injury in diabetic rats

**Fig 3:**
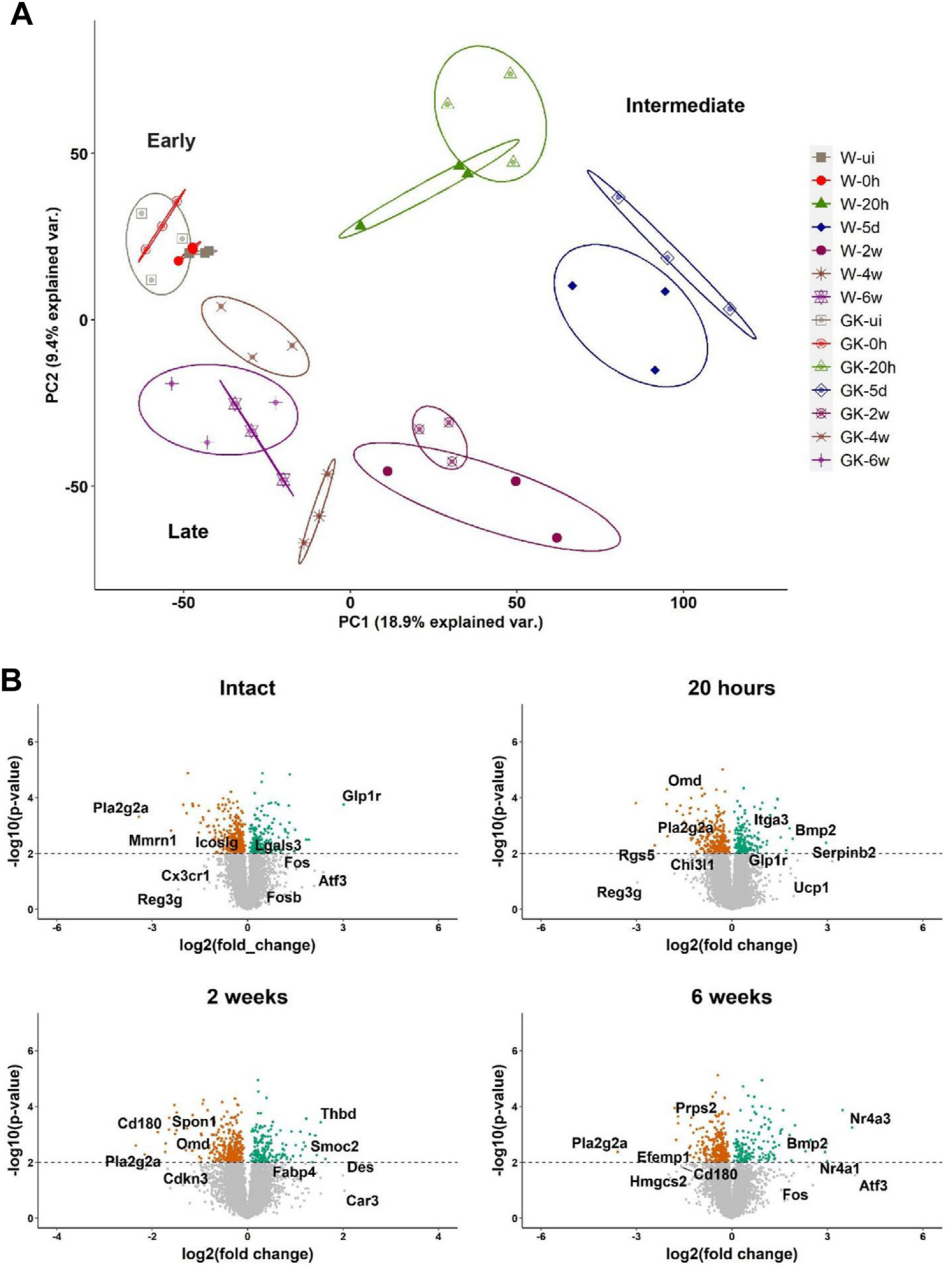
Transcriptomic changes in response to vascular injury in Goto-Kakizaki (*GK*) rats. **A**, Principal component analysis (PCA) of global gene expression profiles of Wistar and GK rats, showing intact and injured vessels collected at early (0 hours), intermediate (20 hours and 5 days), and late phases (2, 4 and 6 weeks) of healing after injury. The timepoints are marked in the colors corresponding to the datapoints. **B**, Volcano plots showing differential gene expression comparing Wistar vs GK rats in uninjured and injured vessels through alternate time points (20 hours, 2 and 6 weeks) during the course of vascular healing. Selected up- and down-regulated genes are highlighted in each plot. The dashed lines represent the nominal *P*-value threshold (*P* = .05).

The global gene expression profiles of Wistar and GK rats from each timepoint were compared individually in a principal component analysis (PCA). The PCA plot showed that only about 28% of the variations in gene expression between the groups were explained by the first two principal components (Fig 3, *A*), indicating that the variation in the data is more complex. The first two principal components did not separate the expression profiles of uninjured and injured vessels at 0 hours in both Wistar and GK rats. However, the expression profiles could be grouped into early, intermediate, and late phases of healing by PCA analysis, as previously reported.^11^ The intermediate phase profile was prominently distinct from the early and late phases of vascular healing. In the late phase of healing, the expression profiles approached closer to the earlier timepoints, indicating the achievement of global equilibrium in gene expression towards the end of healing in the vessels. Interestingly, the gene expression profiles of GK and Wistar rats differed clearly at 4 weeks after injury.

**Supplementary Fig 3:**
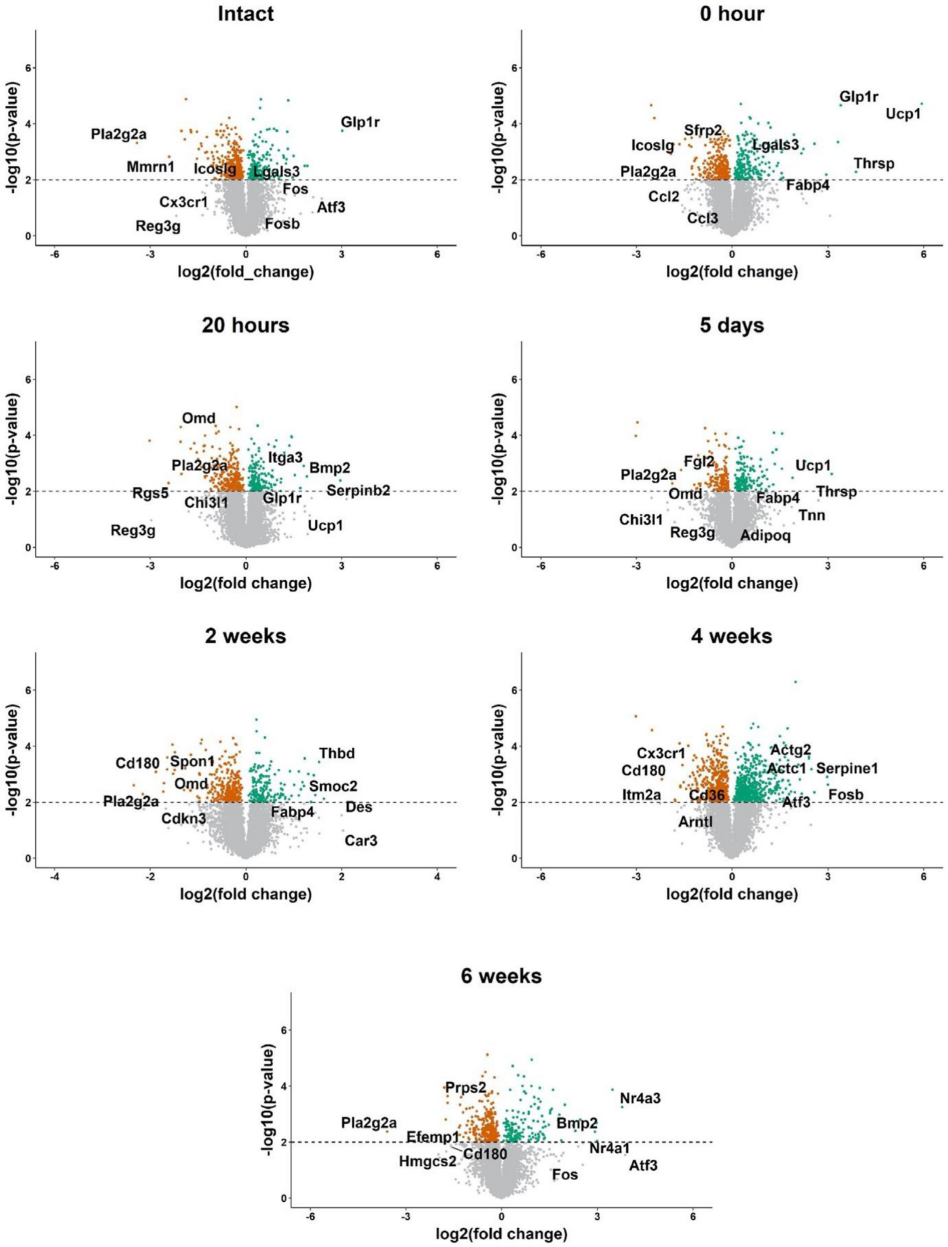
Volcano plots showing differential gene expression comparing Wistar vs GK rats in uninjured vessel and in injured vessel at 0 and 20 hours, 5 days, and 2, 4, and 6 weeks following injury. Selected up- and down-regulated genes are highlighted in each plot. The *dashed lines* represent the nominal *P*-value threshold (*P* = .05).

To further analyze the transcriptomic differences between the groups, differential gene expression comparing Wistar and GK rats was performed for each timepoint as visualized by volcano plots (Fig 3, *B*, Supplementary Fig 3 and Supplementary Appendix). GK rats upregulated genes involved in glucose (Glp1r) and fatty acid metabolism (Thrsp, Fabp4) immediately following injury (0 hour), whereas key immune response molecules such as Pla2g2a, Icoslg, Ccl2, and Ccl3 were downregulated. Genes related to coagulation and cell adhesion were upregulated only at 20 hours (Serpinb2, Itga3). Notably, Bmp2, a negative regulator of SMC proliferation, was also increased at this timepoint. Day 5 after injury was characterized by an increase in genes responsible for lipid metabolism. Coagulation after injury was resolved as late as 2 weeks post injury (Thbd), whereas genes regulating SMC proliferation (Spon1) were still downregulated. At 4 weeks after injury, cytoskeletal and proliferation markers were increased (Actc1, Actg1, Fosb), and immune processes were decreased (Cd36, Cx3cr1, Cd180). Genes regulating proliferation of SMCs were still increased at 6 weeks post injury (Nr4a3, Nr4a1).

### Aberrant SMC and immune cell signaling characterize vessel wall healing in diabetic rats

**Supplementary Fig 4:**
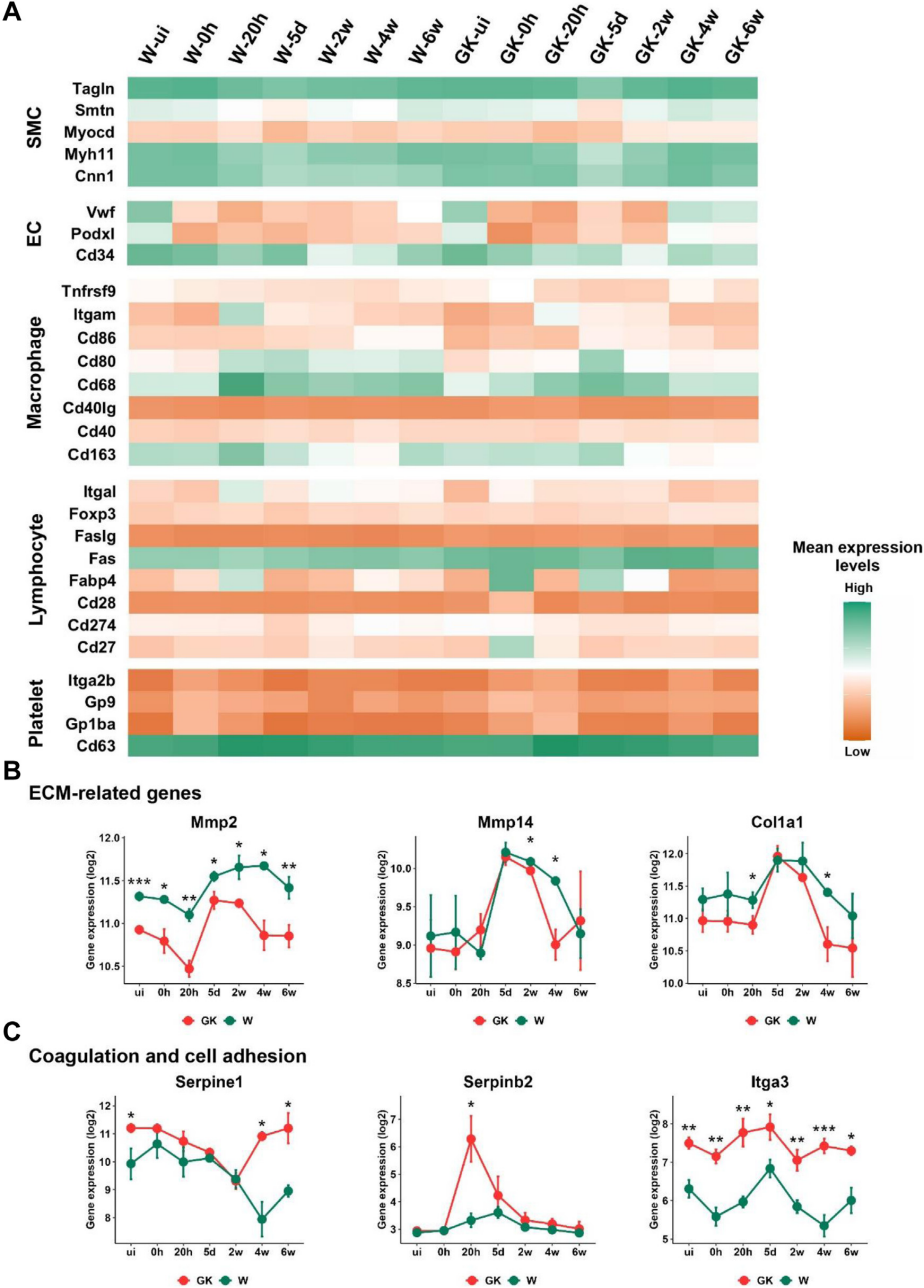
**(A)** A heatmap of mean gene expression levels of cell-specific markers of smooth muscle cells (*SMCs*), endothelial cells (*ECs*), macrophages, lymphocytes, and platelets in Wistar and GK rats in intact and injured vessels across different time points. Line graphs of ECM-related **(B)** and coagulation and cell adhesion-related genes **(C)** in GK rats compared with Wistar rats through all time points. *Green line* indicates Wistar and *red line* indicates GK rats. ∗*P* < .05; ∗∗*P* < .01; ∗∗∗*P* < .001.

The contribution of various major cell types in the vascular wall to vessel healing was assessed from microarray data. Previously recognized classical markers of SMCs, ECs, macrophages, lymphocytes, and platelets were queried for each timepoint in GK and Wistar rats and plotted as a heat map (Supplementary Fig 4).

**Fig 4:**
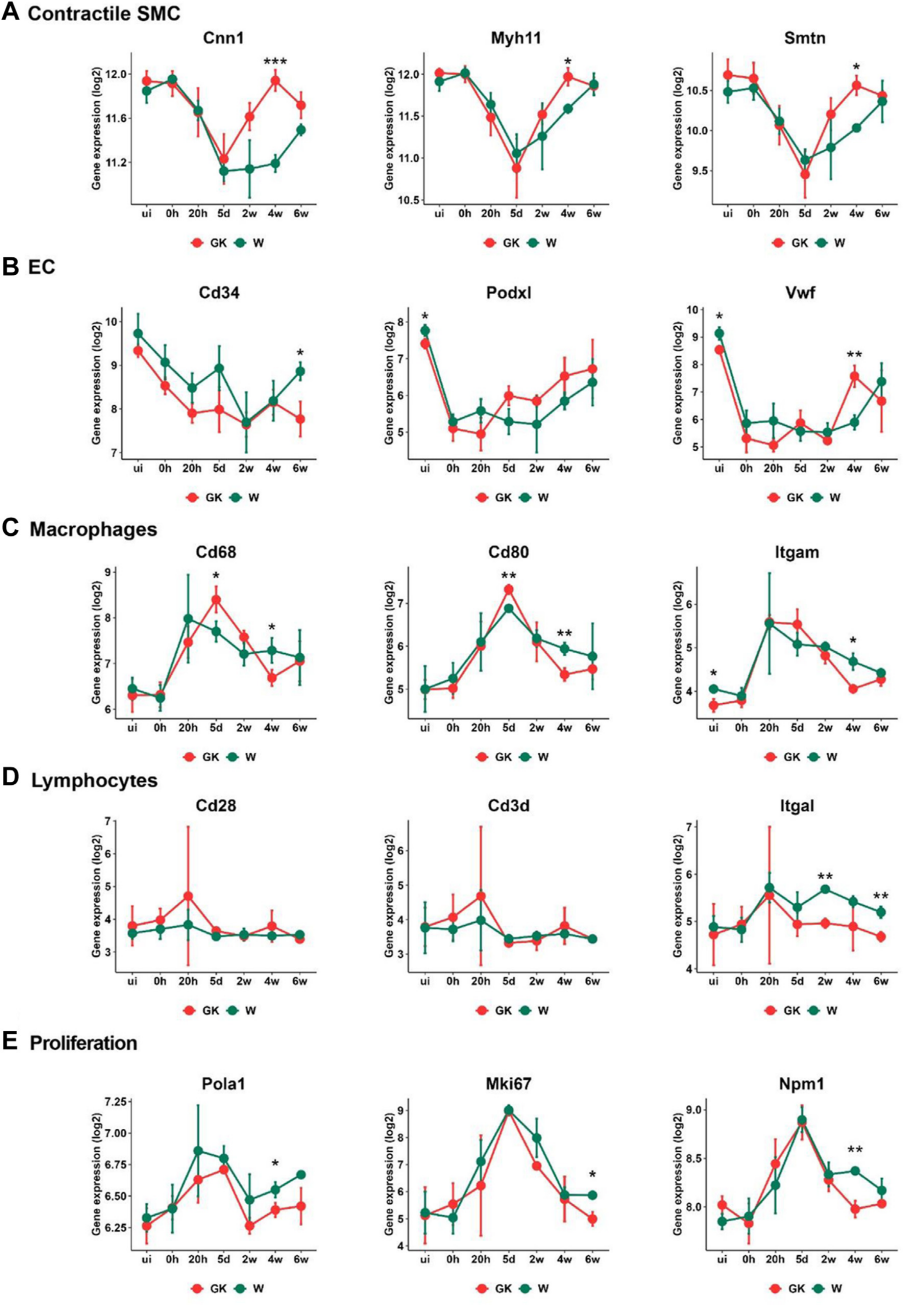
Aberrant smooth muscle cell (*SMC*) and immune cell signaling characterizes diabetic rat vessel wall healing. Line graph of selected genes from different cell types/cell processes in Goto-Kakizaki (*GK*) rats compared with Wistar rats through all time points. *Green line* indicates Wistar, and *red line* indicates GK rats. *EC*, Endothelial cells; *vWF*, von Willenbrand factor. Statistical differences were performed using the Student *t* test for each timepoint between the groups. ∗*P* < .05; ∗∗*P* < .01; ∗∗∗*P* < .001.

As expected, the contractile SMC markers decreased consistently in Wistar and GK rats during early phases up to day 5 of vessel healing (Fig 4, *A*). However, in GK rats in later stages of the response, these markers increased significantly more than in Wistar rats (Fig 4, *A* and Supplementary Fig 4). Concurrently, markers indicating ECM degradation and ECM production, indicating SMC migration and proliferation, were significantly reduced in GK rats (Supplementary Fig 4, *B*). In addition, proliferation markers were also reduced during the remodeling phase in the GK rat vessels (Fig 4, *E*). Taken together, these observations establish that the increase in intimal hyperplasia in GK rats at 4 weeks (Fig 2) is due to the dysfunctional increase in contractile SMCs.

The endothelial markers Vwf and Podxl, decreased following vascular injury in both Wistar and GK rats, while they gradually increased after 2 weeks indicating a trend towards vascular homeostasis. However, Vwf expression was significantly increased in GK rats compared with that of Wistar rats (Fig 4, *B*). The endothelial progenitor cell marker Cd34 also decreased following injury and recovered at the end of the healing process in Wistar rats. In GK rats, the expression levels decreased at 6 weeks, indicating a defect in the production of endothelial progenitor cells (Fig 4, *B*).

The macrophage marker Cd68, which peaked at 20 hours in Wistar rat vessels, peaked at 5 days in GK rats, with a significantly increased expression. Cd80, on the other hand, peaked at 5 days in both groups, but was also higher in GK rat vessels. Both these markers fell sharply in GK rats thereafter, decreasing more significantly than Wistar rats at 4 weeks (Fig 4, *C*). Most lymphocyte genes increased at 20 hours after injury in both GK and Wistar rats (Fig 4, *D*). However, the costimulatory molecule Itgal markedly decreased during 2 weeks in GK rats (Fig 4, *D*).

Increase in platelet expression was limited to 0 and 20 hours in GK and Wistar rats (Supplementary Fig 4, *A*). Interestingly, genes that promote coagulation, such as Serpine1 and Serpinb2, had distinctly higher expression in GK rats at various phases of healing (Supplementary Fig 4, *C*). Although Serpine1 was increased in uninjured GK rats' vessels and 4- and 6-weeks injured vessels, Serpinb2 was abnormally increased at 20 hours post injury. The key adhesion molecule Itga3 was higher in uninjured GK rat vessels and was also consistently higher throughout the healing process (Supplementary Fig 4, *C*).

Taken together, these results show that SMC and macrophage gene expression are profoundly affected in GK rats compared with Wistar rats.

### Pathway analyses show that cellular processes central to vascular healing are delayed in diabetic rats

**Fig 5:**
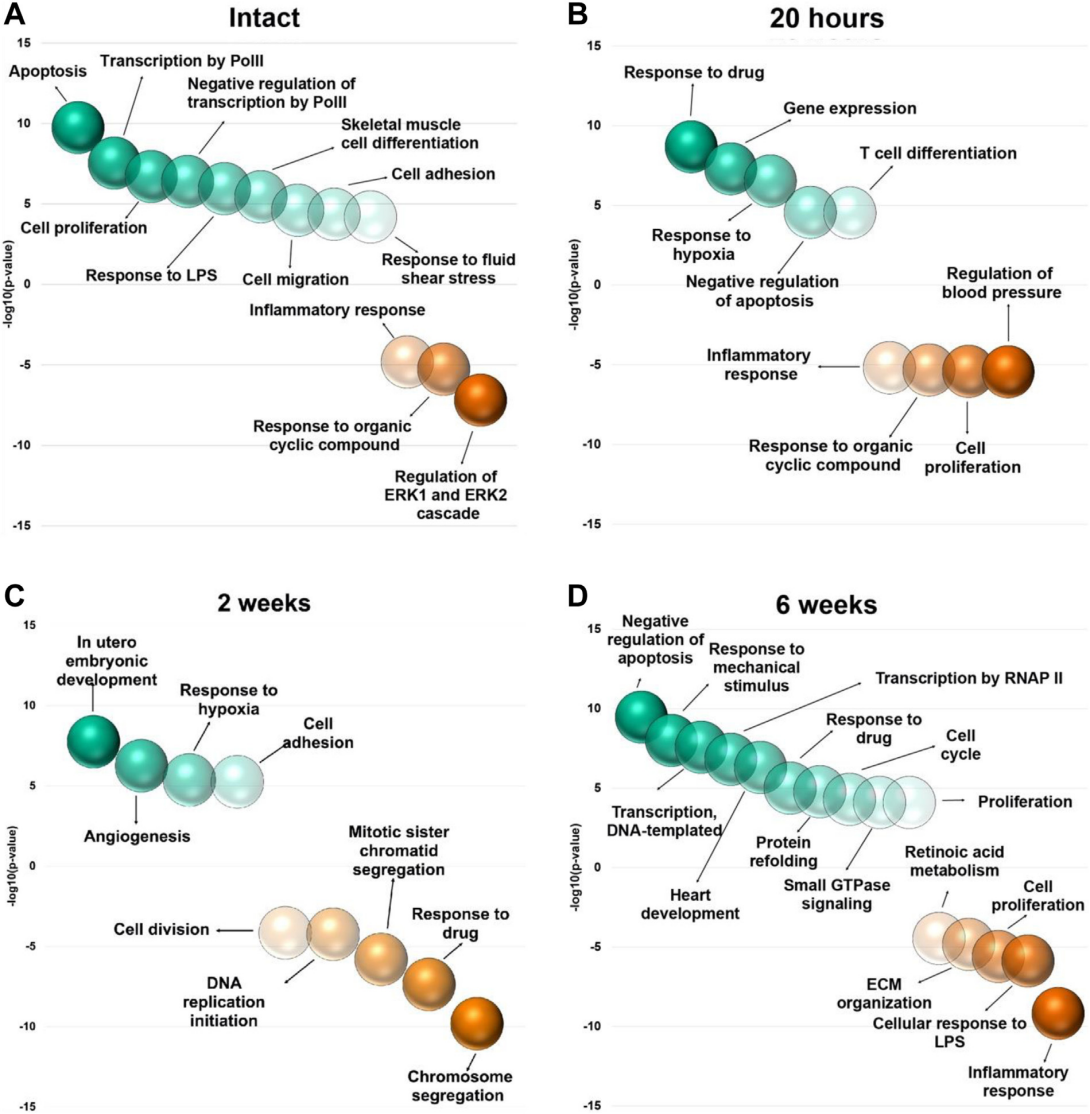
Pathway analyses show that cellular processes central to vascular healing are delayed in diabetic rats. Gene ontology (GO) enrichment analysis was performed for differentially up- and down-regulated genes from different time points (intact, 20 hours, 2 and 6 weeks) after injury. Goto-Kakizaki (GK) rats are characterized by impaired inflammatory response in the early phase of vascular healing, aberrant extracellular matrix (ECM) remodeling and a delayed but protracted induction of cell proliferation. *Green and red bubbles* represent up- and down-regulated pathways, respectively. The increasing color gradient indicates the increasing *P*-value. Only significant pathways with *P* < .05 are shown. *ERK*, Extracellular signal-regulated kinases; *LPS*, lipopolysaccharide; *RNAP II*, RNA polymerase II.

**Supplementary Fig 5:**
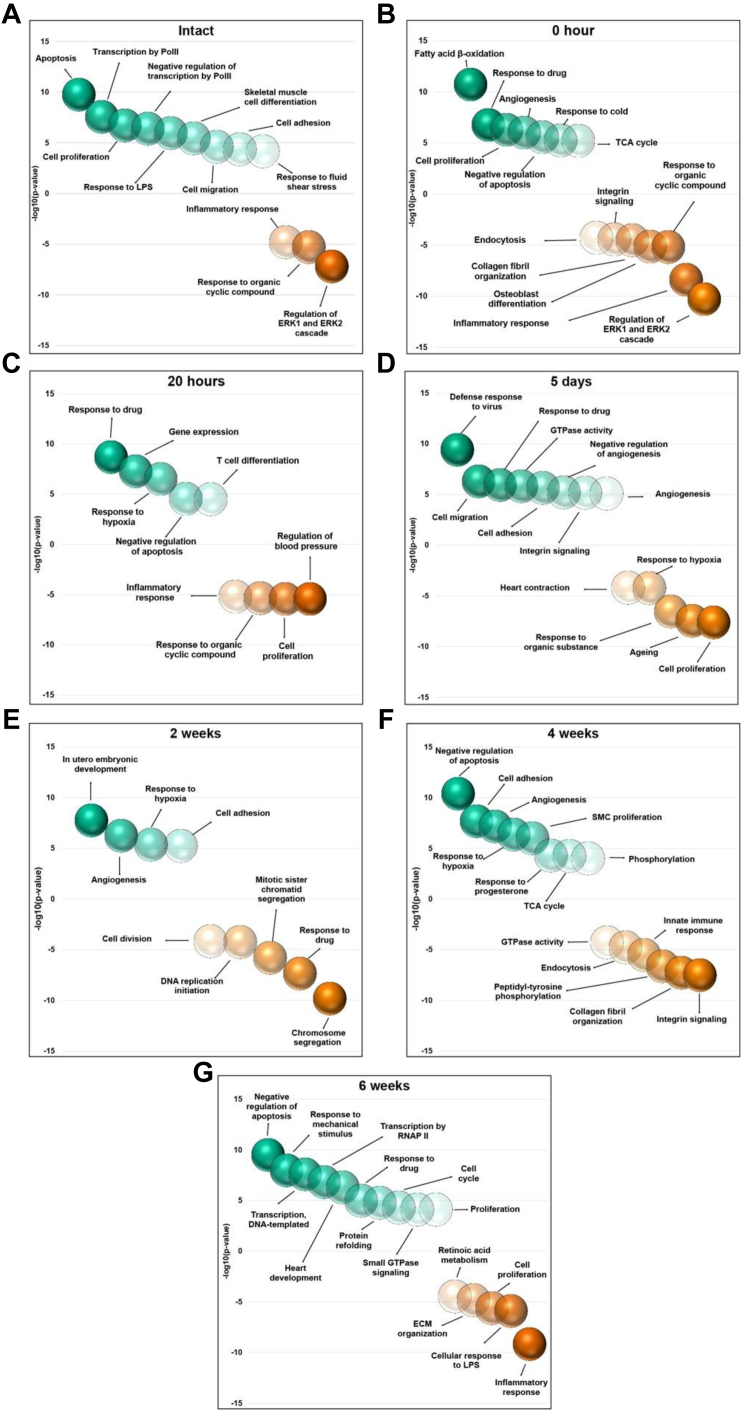
Gene Ontology (GO) enrichment analysis was performed for differentially up- and down-regulated genes from different all points (intact, 0 and 20 hours, 5 days, and 2, 4, and 6 weeks) after injury. *Green and red bubbles* represent up- and down-regulated pathways respectively. The increasing color gradient indicates the increasing *P*-value. Only significant pathways with *P* < .05 are shown. *ECM*, Extracellular matrix; *ERK*, extracellular signal-regulated kinases; *LPS*, lipopolysaccharide; *RNAP II*, RNA polymerase II; *TCA*, tricarboxylic acid.

To get an insight into the consequences of the aberrant signaling in SMCs and immune cells towards overall vessel healing in GK rats, we used the significantly up- and down-regulated genes in GK rats to identify biologically enriched pathways by GO analysis. Certain pathways such as hypoxia signaling and angiogenesis were enriched immediately following injury and persisted until 4 weeks post injury (Supplementary Fig 5). In the early phases of healing (0 and 20 hours), vessels from GK rats were enriched for fatty acid oxidation, hypoxia signaling, cell proliferation and survival, and TCA cycle, indicating overall changes in metabolism. Notably, early phase processes of vessel healing such as inflammatory response and integrin signaling were repressed (Fig 5, *B* and Supplementary Fig 5, *B-C*). In the intermediate phase, processes such as response to virus, integrin signaling, cell adhesion, and migration were enriched, indicating a delayed induction of inflammatory response. However, processes related to cell proliferation were repressed in this phase (Fig 5, *C* and Supplementary Fig 4, *D-E*). The late phase of healing was characterized by SMC proliferation, cell survival, response to hormonal signaling, and response to mechanical stress. It is important to note that processes related to vessel remodeling, such as collagen fibril organization, ECM organization, and integrin signaling, were still repressed (Fig 5, *D* and Supplementary Fig 5, *F-G*). Overall, the pathway analysis corroborated the gene expression changes and confirmed the aberrant induction of inflammatory response and SMC contraction.

### Transcription factor-protein-protein interaction networks (TF-PPI) reveal key pathway modulators in diabetes

**Fig 6:**
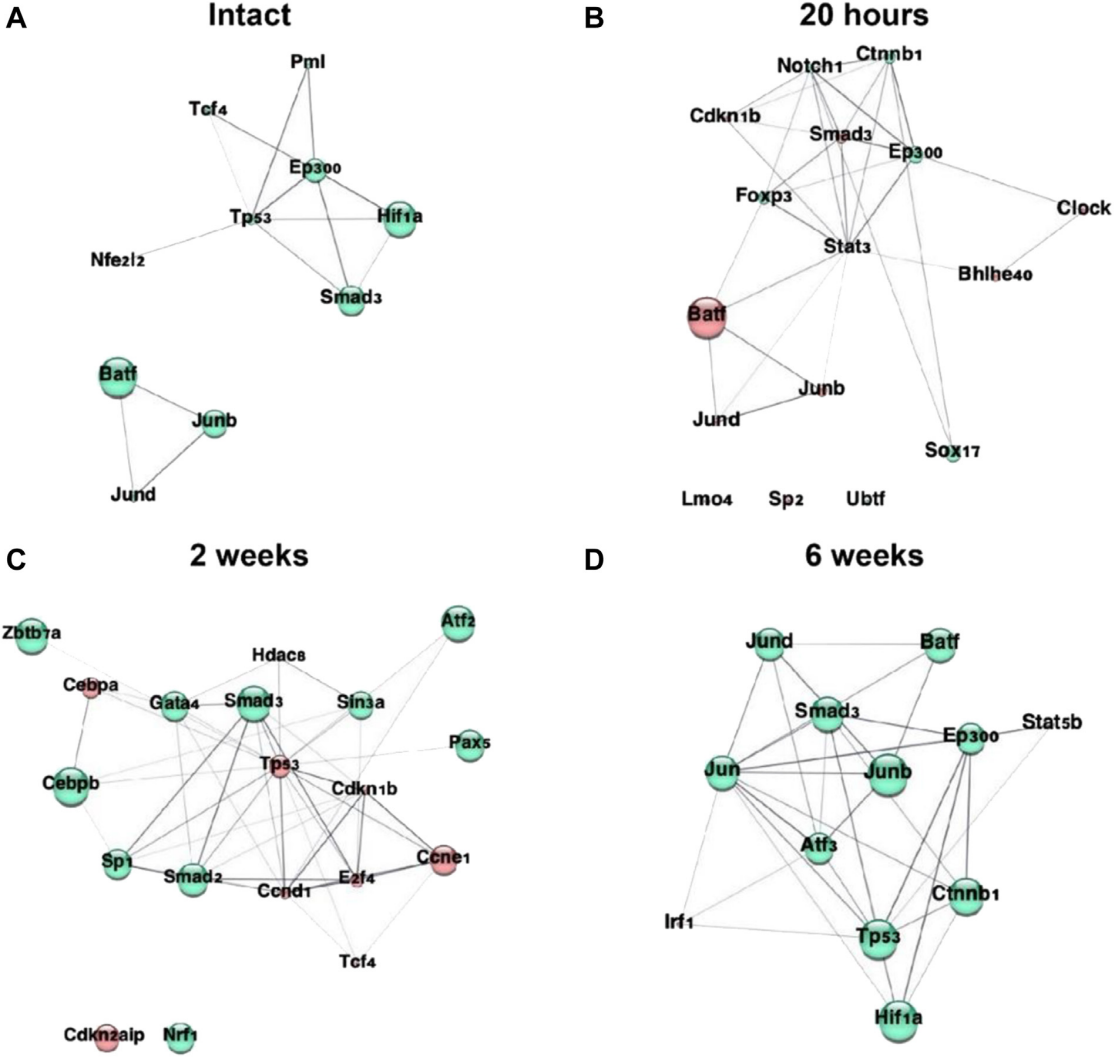
Transcription factor-protein-protein interaction networks (TF-PPI) key pathway modulators in diabetes. A network of significantly modulated TF-PPIs for intact **(A)** and injured vessels at different timepoints - 20 hours **(B)**, 2 weeks **(C),** and 6 weeks **(D)**. Significantly up- and down-regulated genes from each timepoint comparing Goto-Kakizaki (GK) vs Wistar rats, were used to obtain TF-PPI, and this information was fed into STRING database to generate the network. The top 10 up- and 10 down-regulated TFs are shown in the network above. Up- and down-regulated TFs are indicated in *green* and *red* nodes respectively. Size of the nodes indicate the levels of *P*-value. All the interactions were predicted with the adjusted *P*-value < .05.

**Supplementary Fig 6:**
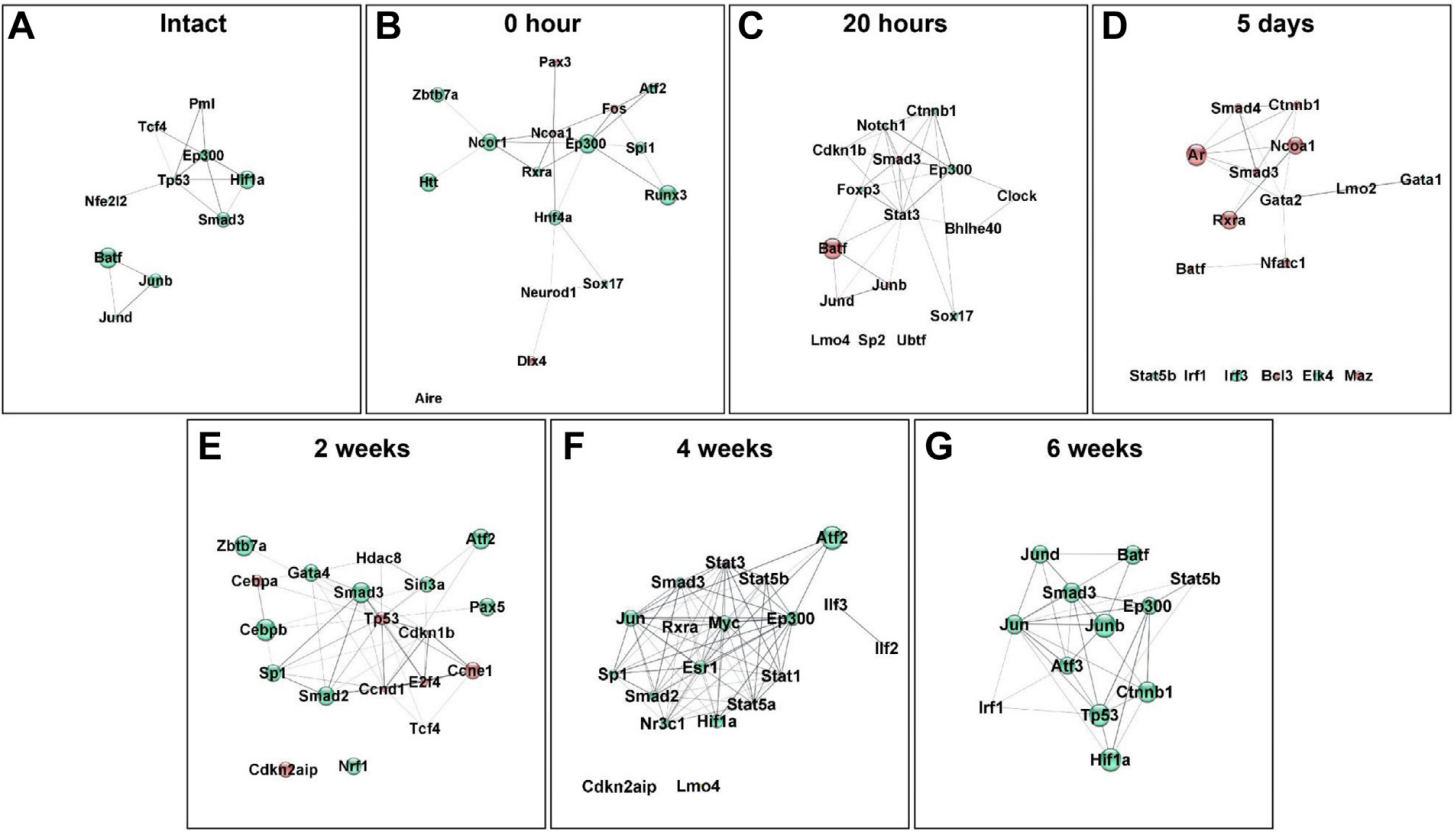
Top 10 significantly modulated TF-PPIs in GK rats compared Wistar rats for intact **(A)** and injured vessels at different time points: 0 hours **(B)**, 20 hours **(C)**, 5 days **(D)**, 2 weeks **(E)**, 4 weeks **(F)**, and 6 weeks **(G)**. Significantly up- and down-regulated genes from each time point were used to obtain TF-PPI and this information was fed into STRING database to generate the network. The top 10 up- and 10 down-regulated TFs are shown in the network above. Up- and down-regulated TFs are indicated in *green and red nodes* respectively. Size of the nodes indicate the levels of *P*-value. All the interactions were predicted with the adjusted *P*-value < .05.

To identify regulatory changes that potentially led to dysregulated vessel healing in GK rats, we performed analysis of TF-PPI interaction networks. The analysis showed that following injury, genes responsible for induction of proliferation were repressed at several levels, with upregulation of transcriptional repressors such as Zbtb7a, Ncor1, and Sox17 and with the repression of transcriptional activators such as Ncoa1, Fos and Neurod1 (Supplementary Fig 6, *B*). At 20 hours, TFs that induce proliferation and differentiation were upregulated (Ctnnb1, Ep300, Notch1). However, induction of Foxp3 and downregulation of Batf, Stat3, and Smad3 indicated a dampening of inflammatory response at this early stage of vessel wall healing (Fig 6, *B*). Increase in Gata1, Gata2, and Lmo2 expression signaled the beginning of the endothelium recovery, whereas inflammatory responses were still repressed (Batf, Nfatc1, Smad3, Rxra) after 5 days of injury (Supplementary Fig 6, *D*). The immune response to the injury was finally induced at 2 weeks with the increase of Sp1, Cebpb, Pax5, and Smad3. However, transcription factors that promote T-cell differentiation were suppressed through 20 hours (Stat3, Batf) and 5 days (Nfatc1, Batf, Ncoa1) and activated only at 2 weeks (Pax5, Sin3a, Cebpb) and continued through 4 weeks (Sp1, Jun, Nr3c1) and 6 weeks (Batf, Jun). It is important to note that, at this point, cell proliferation is strongly repressed by the downregulation of many cell cycle genes (Ccnd1, Ccne1, E2f4) and upregulation of transcriptional repressors (Zbtb7a, Atf2) (Fig 6, *C*). At 4 weeks, these immune responses diminished as shown by the activation of Smad3, Jun, and Nr3c1 and repression of Stat3 and Stat5a. However, various other cell survival and proliferation markers were also activated (Hif1a, Myc, Atf2, Ep300) (Supplementary Fig 6, *F*). This trend continued at 6 weeks as well, since proliferation markers were consistently upregulated (Junb, Jund, Jun, Ep300, Atf3, Ctnnb1) (Fig 6, *D*). In summary, the network of TF-PPIs at each timepoint confirmed that the typical healing responses were delayed in GK rats, and this dysregulation was controlled at the transcriptional level.

## Discussion

In this study, we investigated temporal transcriptomic changes in the vessel wall that drive faulty arterial recovery in diabetic rats. Ultrasound analysis revealed that GK rats had an increased intimal hyperplasia in parallel with exaggerated negative remodeling, which was confirmed by histological analyses. Global transcriptomic analysis of the vessels in GK and Wistar rats revealed changes in expression patterns, which could explain the altered physiological and morphological phenotype.

The PCA analysis showed that the biggest changes in the transcriptomic profiles of Wistar and GK rats were observed at 20 hours, 5 days, 2 weeks, and 4 weeks, with most profound differences at 4 weeks following injury, corresponding to the late stages during the remodeling phase. This was corroborated by the US and histological analysis, where lumen was significantly smaller in GK rats at 4 and 6 weeks after injury compared with Wistar rats.

On the level of individual genes, classical contractile SMC markers decreased equally in both groups during the acute injury phase, but the increase in the remodeling phase in GK rats was much higher compared with Wistar rats. This observation is strengthened by the appearance of contractile markers and SMC-specific transcription factors as the top upregulated genes at 4 and 6 weeks. Indeed, it is known that luminal narrowing during early part of the healing is due to smooth muscle contraction.^18^ However, the increased SMC contraction in GK rats even after 2 weeks post injury could potentially be the source of the exaggerated negative remodeling. Indeed, previous reports have shown that increased production of ROS could increase the contractile gene expression in the aorta of GK rats.^9^ At the same time, the expression of matrix metalloproteinase (MMP)-2 and MMP-14 was significantly decreased during the remodeling phase, along with an increase in Collagen type IV and a decrease in Collagen type I. It is well known that the MMP-14 activates MMP-2, which, in turn, is required to cleave Collagen type IV in order to facilitate SMC motility.^10^, ^11^, ^12^ These observations show that impairment in MMP activity may potentially lead to decreased motility and proliferation of SMCs during vascular healing.

Pathway analysis showed an upregulation of SMC proliferation at 4 weeks, further confirmed by the induction of transcription factors that promote SMC proliferation^19^, ^20^, ^21^ from 2 weeks after injury. Our finding is in line with previous reports that intimal hyperplasia in patients with diabetes is phenotypically different, and SMCs from diabetic vasculature have greater adhesion, migration, and proliferation potential.^4^^,^^22^ Interestingly, glucose-lowering agents have been reported to inhibit SMC proliferation and improve restenosis in patients with diabetes.^23^

The endothelial markers decreased, as expected, following injury to vessel wall, and gradually increased in expression after 2 weeks as the vessel wall approached homeostasis, in both GK and Wistar rats. However, von Willebrand factor (vWF), which is also a key molecule for platelet aggregation after tissue injury, was lower in intact vessels of GK rats, suggesting a possible endothelial dysfunction at baseline. As their expression levels increased towards the late phase of healing, there was a significant increase in vWF levels in GK rats at 4 weeks compared with Wistar rats. It is also noteworthy that the GK rats had an increased amount of thrombosis after surgery. On the contrary, the carotid diameter of the GK rats were relatively smaller compared with Wistar rats, which could also have contributed to increased thrombosis. The increase in vWF levels coincided with the specific increase of all platelet markers at 4 weeks in GK rats. Abnormal platelet activation is a feature of diabetes, and, in the context of restenosis, could lead to thrombosis in patients with diabetes.^24^ Indeed, vWF is reported to mediate SMC proliferation in intimal hyperplasia,^25^ and it is tempting to speculate that increased vWF expression in the GK rat vessels might be one of the contributing factors to increased SMC proliferation. We found irregular increases in gene expression of molecules controlling cell adhesion and coagulation at various stages of healing. Increase in plasminogen activator inhibitor type 1 (PAI-1), the protein product of the Serpine1 gene, results in thrombosis and SMC proliferation, and has been reported to be associated with hyperinsulinemia.^26^ In addition, a decrease in endothelial progenitor cell (EPC) marker, at 6 weeks in GK rats, indicated an impairment in EPC recruitment to the site of injury, a common feature of diabetes-induced dysfunctional neovascularization.^27^ Taken together, these changes in gene expression provide molecular evidence for the impaired reendothelialization and higher rate of thrombosis observed in GK rats.

We observed an abnormal induction of innate and adaptive immune response during vascular healing in GK rats. Particularly, in the late phases of healing (at 4 weeks), macrophage markers reduced significantly in GK rats compared with Wistar rats. The lymphocyte marker expression did not change in GK rats. However, reduction in Itgal levels, but not Cd28 levels, shows that in GK rats, the adhesion of T cells to antigen presenting cells seem defective.^28^ In summary, the healing in GK rats is delayed due to a protracted and overactivated innate immune response.

Restenosis is characterized by a chronic inflammatory response and negative remodeling with SMC proliferation due to delayed re-endothelialization. Our observations in this study confirm these features are recapitulated in the balloon catheter injury of carotid arteries in diabetic rats. Future studies into the mechanisms of the dysfunctional build-up of SMCs during the remodeling phase and the aberrant regulation of macrophages throughout the injury could provide further insights into the pathogenesis of restenosis in patients with diabetes.

### Limitations and advantages

It is important to note that, although GK rats generally have moderate increase in blood glucose levels, they also have relatively normal or moderately decreased insulin levels. This is because the changes in insulin levels are caused only by the decrease in β-cell mass and are not driven by obesity.^13^ However, GK rats resemble the causal mechanism of type 2 diabetes in humans, and thus, have an increased value for studying restenosis in such a context.

It is also important to note that the study was conducted exclusively in male rats. Studies focused on investigating gender differences in restenosis have found that females have a significantly lower incidence of in-stent restenosis.^1^ In addition, females in general present lower risk for cardiovascular disease due to the protective effect of estrogen on the vascular wall.^2^ On the other hand, while diabetes significantly increases the risk of both early and in-stent restenosis in females,^1^ inclusion of females to the study would require specific experimental design which is above the scope of this study. More efforts need to be made to elucidate the effect of sex on the vascular healing in diabetes.

When it comes to some advantages, this study can be seen as a global longitudinal encyclopedia of dysregulated arterial healing in diabetes, generating a powerful resource available for future explorations in this field. Moreover, the integration of morphological data from minimally invasive US with histological and transcriptomic data allows for direct correlation between the physiology of the vascular wall and molecular changes occurring in various cell types during the healing, thus improving the robustness of the observations.

## Conclusions and perspectives

We systemically analyzed the morphological and physiological changes during vascular healing at various timepoints, together with longitudinal transcriptomic changes and their molecular drivers in diabetic GK rats. A striking difference was observed in the morphology and delayed healing in response to injury between GK and Wistar rats. Transcriptomic analysis revealed that, although Wistar rats had a healing response similar to that reported previously in healthy Sprague Dawley rats,^11^ GK rats had an altered gene expression pattern characterized by increased SMC contraction, platelets activation, delayed and prolonged inflammatory response, and dysfunctional reendothelialization. Specifically, the impaired control of SMC proliferation was associated with a decrease in lumen area at later timepoints post injury, whereas other altered processes could be viewed as contributing factors to SMC and EC dysfunction during vessel healing in diabetes. More dedicated studies about the contribution of hyperglycemia to the SMC contraction and other disturbed pathways found here could be helpful in understanding the development of restenosis, and also to identify novel effective treatment opportunities for patients with diabetes following vascular interventions.

## Author Contributions

Conception and design: SR, LM, AR

Analysis and interpretation: SN, ML, MK

Data collection: SR

Writing the article: SN

Critical revision of the article: SN, SR, ML, MK, LM, AR

Final approval of the article: LM, AR

Statistical analysis: SN

Obtained funding: LM

Overall responsibility: AR

LM and AR contributed equally to this article and share co-senior authorship.
